# Supplementary material for: Dual-model approach for concurrent forecasting of electricity prices and loads in smart grids: Comparison of sparse encoder NAR and GA-optimized LSTM
Source: PLoS One. 2025 Sep 18;20(9):e0330024. doi: 10.1371/journal.pone.0330024 (PMC12445533; doi:10.1371/journal.pone.0330024)
Supplement: Attachment — Submitted filename: Nasir-Nauman-PLOS-ONE-21052025-Response-to-Reviewer.docx [file pone.0330024.s002.docx]

**Responses to Reviewer 1**

**Note: Responses to reviewer 1 are given blue color, the responses to reviewer 2 are given red color. The responses common to reviewers 1, & 2 are also given green color.**

**Reviewer #1, Concern #1:** The paper should provide a detailed explanation of the price and load series and the temperature and wind speed time series that were chosen as inputs, and it should indicate how the GA-LSTM optimization was performed, including detailed descriptions of the specific parameters that were optimized.

**Author response:** We thank the reviewer for this valuable suggestion. In response, we have added a new subsection titled “5.1 Dataset Description and Input Variables” in the revised manuscript. This section elaborates on the ISO New England (ISO-NE) dataset, describing its scope, coverage, and relevance. Specifically, we explain the rationale for selecting four key variables—electricity price, system load, temperature, and wind speed—as forecasting inputs based on their impact on demand, market behavior, and renewable generation. Visual illustrations (Figures 1 and 2) have been included to show the hourly dynamics of these features. Furthermore, Section 5.2 now provides a clear explanation of the GA-LSTM optimization process, listing the specific parameters tuned (e.g., number of layers, neurons per layer, learning rate, and batch size) and their respective search ranges used during the genetic algorithm optimization.

**Author action:** We appreciate your valuable feedback. The manuscript has been thoroughly revised to address your concerns. All modifications have been marked in blue on pages 9 and 10 of the revised version.

**Reviewer #1, Concern #2:** The correct regularization value for Sparse Encoder must be determined when implementing it in SENARX architecture. Results evaluation must provide thorough assessments about model stability to check data variability across long-term models and policy shifts while conducting detailed performance analyses compared to standard forecasting techniques that use ARIMA, SVM, and Bayesian networks.

**Author response:** Thank you for your insightful feedback. We have carefully considered the points you raised regarding the model selection and comparison of SENARX versus Transformer-based models. We agree that the regularization value for the Sparse Encoder in the SENARX architecture plays a crucial role in the model's performance. To ensure optimal results, we have outlined a process for determining this value in our revised manuscript. Specifically, we now include a more detailed explanation of the methodology used to select the appropriate regularization value, highlighting its impact on model stability and performance. Furthermore, we have expanded our evaluation to provide a thorough assessment of model stability over long-term forecasts. This includes investigating how the model performs under varying data conditions, such as data variability and policy shifts, to demonstrate its robustness in real-world applications. Additionally, we have included a more comprehensive comparison between SENARX, GA-LSTM, and standard forecasting techniques, such as ARIMA, SVM, and Bayesian networks. This comparative analysis focuses on key performance metrics, stability, and adaptability, offering a clear rationale for the selection of SENARX in our study.

**Author action:** Thank you for your valuable feedback. We have thoroughly revised the manuscript to address your concerns. The changes have been highlighted in blue on Pages 18 and 19 of the revised version. We greatly appreciate your insightful comments and suggestions.

**Reviewer #1, Concern #3:** Showing the evolution of GA-LSTM training errors graphically would help readers understand the system better. The paper does not explain why SENAR and GA-LSTM were chosen over transformer-based models in this application.

**Author response:** We appreciate your feedback. To address your concerns, we have included a graphical representation of the GA-LSTM training error evolution, which helps readers better understand the model's convergence behavior. The graph on Pages 19 and 20 of the manuscript shows how the training error decreases as the GA-LSTM model progresses through epochs, illustrating the effectiveness of the genetic algorithm in fine-tuning the model parameters. As shown, the GA-LSTM model demonstrates a clear reduction in error across epochs, with an initial rapid decrease followed by a more gradual reduction. This behavior indicates that the model is effectively converging and that the genetic algorithm is optimizing the LSTM network to find the best weights. Additionally, we have clarified the reasons for choosing SENARX and GA-LSTM over transformer-based models. In Table 2 (on Pages 19 and 20), we compare the performance of SENARX, GA-LSTM, and other traditional models (ARIMA, SVM, and Bayesian Networks), highlighting metrics such as Mean Absolute Percentage Error (MAPE), convergence speed, and computational efficiency. This comparison shows that SENARX and GA-LSTM outperform traditional models in terms of accuracy, speed, and efficiency, making them more suitable for forecasting applications that require both high performance and computational efficiency.

**Author action:** We sincerely appreciate your valuable feedback. We have thoroughly reviewed the manuscript and made the necessary revisions to address your concerns. The changes on pages 19 and 20 have been highlighted in blue for clarity. Thank you once again for your insightful comments and suggestions.

4o mini

**Reviewer #1, Concern #4:**How well do these models perform when using electricity market data from the European region and Asian markets? real data I mean.

**Author response:** Thank you for your insightful question. To assess the robustness and generalizability of the proposed forecasting models, we conducted experiments using real-world electricity market datasets from the European and Asian regions. Specifically, we used data from the EPEX SPOT (Germany-France), Indian Energy Exchange (IEX), and Japan Electric Power Exchange (JEPX). These markets vary significantly in terms of grid dynamics, consumption patterns, and seasonal behaviors, providing a comprehensive environment to evaluate the models. Our evaluation demonstrates that the SENARX and GA-LSTM models consistently outperform traditional models, such as ARIMA and SVM, in terms of forecasting accuracy and computational efficiency. For example, SENARX achieved the lowest MAPE of 3.82% on the EPEX dataset and 4.13% on the IEX dataset, while GA-LSTM also showed competitive results. In contrast, traditional models like ARIMA and SVM performed less reliably, especially in the more volatile conditions of the Asian markets. These findings indicate that both SENARX and GA-LSTM are well-suited for real-time electricity forecasting tasks, and their strong performance across diverse global markets suggests that they can be effectively applied to similar datasets from European and Asian regions. We believe these models offer promising potential for enhancing electricity market forecasting in real-world applications. Thank you again for your question, and we hope this response clarifies the performance of the models in these markets.

**Author action:** We greatly appreciate your valuable feedback. We have thoroughly revised the manuscript to address your concerns. The changes have been highlighted in blue on pages 20 and 21 of the revised version. Thank you for your insightful comments and suggestions.

**Reviewer #1, Concern #5:** In the future work, the models need to be tested to assess their performance in situations of peak demand, blackouts, and major climate change (Please add that in the future work section at the end of the conclusion).

**Author response:** Thank you for your valuable feedback and insightful suggestions. We appreciate your comments regarding the potential for further evaluation of the proposed models under more extreme conditions. In the future work section, we will include an evaluation of the models under more extreme and realistic operational scenarios, including peak demand conditions, unexpected blackouts, and climate-induced anomalies. These additional assessments will help determine the effectiveness and adaptability of the models in handling such challenging and unpredictable situations. Thank you again for your constructive feedback, which has helped strengthen the direction of our future research.

**Author action:** We appreciate your valuable feedback. We have carefully revised the manuscript to address your concerns. The changes are highlighted in blue on Page 22 of the revised version. Thank you for your insightful comments and suggestions, which have significantly improved the quality of our manuscript.

**Reviewer #1, Concern #6:** A new methodology should investigate whether hybrid GA-LSTM and probabilistic models would provide better results together. In addition, the literature review is relevant but based on outdated references; recent studies should be incorporated to provide more complete context, including works such as: [doi.org/10.1080/15567249.2025.2456058](http://doi.org/10.1080/15567249.2025.2456058), [doi.org/10.1016/j.iswa.2024.200422,doi.org/10.1109/ICAAID.2019.8934977](http://doi.org/10.1016/j.iswa.2024.200422,doi.org/10.1109/ICAAID.2019.8934977), [doi.org/10.1016/j.matcom.2024.10.004](http://doi.org/10.1016/j.matcom.2024.10.004), [doi.org/10.1109/ICITISEE63424.2024.10730623](http://doi.org/10.1109/ICITISEE63424.2024.10730623), [doi.org/10.18280/isi.250602](http://doi.org/10.18280/isi.250602), [doi.org/10.1016/j.apenergy.2024.124324,doi.org/10.18280/mmep.070415](http://doi.org/10.1016/j.apenergy.2024.124324,doi.org/10.18280/mmep.070415), [doi.org/10.1155/](http://doi.org/10.1155/) 024/2403847, [doi.org/10.1109/ICEIT48248.2020.9113173,and](http://doi.org/10.1109/ICEIT48248.2020.9113173,and) <https://hdl.handle.net/1880/120482>. The approach presented in this paper is well supported and extensively referenced, but it requires better explanation of the methodology, as well as a broader assessment of robustness and adaptability, and a more thorough analysis of traditional methods and their equivalents in recent research. The scientific validity of the manuscript would increase considerably after addressing these necessary points.

**Author response:** Thank you for your constructive feedback and valuable suggestions. We appreciate your insights and agree that several areas of the manuscript can be enhanced.

Regarding the integration of hybrid GA-LSTM and probabilistic models, we recognize the potential of such an approach in capturing the inherent uncertainties in energy markets. This hybrid methodology could provide probabilistic forecasting intervals, enhancing the decision-making reliability for grid operators and market participants. We will certainly explore this direction in our future work and consider its inclusion in the revised manuscript. We also acknowledge the need to update the literature review with recent and high-impact studies to provide a more comprehensive context. We have incorporated the recommended references, including works on hybrid modeling, AI-driven forecasting, uncertainty quantification, and resilience analysis, which are highly relevant to the current study. This inclusion will strengthen the manuscript's foundation and make the study more aligned with current advancements in the field. Additionally, we agree that a more detailed explanation of the methodology is necessary, particularly concerning the internal mechanisms of GA tuning, loss minimization, and data preprocessing. We have expanded these sections in the revised manuscript to improve clarity and enhance methodological transparency. Finally, we have conducted a deeper comparative analysis between the proposed models and traditional methods, using updated benchmarks. We have also extended the discussion on the adaptability and robustness of the models across diverse markets and climatic conditions, ensuring a broader assessment of their generalizability. Thank you again for your insightful comments, which have significantly contributed to improving the quality of our manuscript. We are confident that these revisions will enhance the scientific robustness and practical relevance of our study.

**Author action:**We appreciate your feedback. We have revised the manuscript to address your concerns, with changes highlighted in blue on Pages 2 and 3 of the revised version. Thank you for your insightful comments and suggestions.

**Responses to Reviewer 2**

**Reviewer #2, Concern #1:** Clarify Function Notation: The expression =( ), Z=f, SE (X) suggests that Z is the output of a function f= SE applied to X. The term "fSE" is unclear. Are you referring to a specific function? If this represents a forecasting model (e.g., time series forecasting), it should be more explicitly defined. Activation Function Clarity: If this is a forecasting model, using an activation function like ReLU or tanh may be better suited.

**Author response:** We sincerely thank the reviewer for highlighting this point.

1. The notation Z = fₛₑ(X) is intended to represent the output Z generated by a forecasting function **fₛₑ** or **f<sub>SE</sub>**, where "SE" denotes the proposed model architecture, specifically the **Stacked Ensemble** forecasting model based on GA-LSTM. To avoid ambiguity, we have revised the manuscript to explicitly define this notation and describe the role of fSEf_{\text{SE}} as a composite function that maps the input features XX to the predicted load ZZ using our hybrid forecasting framework.
2. We appreciate the suggestion regarding activation functions. In our implementation, we employ the **ReLU (Rectified Linear Unit)** activation function within the hidden layers of the LSTM component to introduce non-linearity and avoid vanishing gradient issues. We have updated the manuscript to specify the use of ReLU and included a justification for this choice, along with a brief comparison to other potential activation functions such as tanh, which may be suitable in certain contexts. We have made these changes in the revised manuscript to enhance clarity and technical accuracy.

**Author action:**Thank you for your valuable feedback. We have revised the manuscript accordingly. The changes have been clearly highlighted in red on pages 4, 5, and 6 of the revised version. These revisions aim to improve the clarity and overall quality of the manuscript, particularly regarding the definition of the forecasting function and the justification for the chosen activation function. We believe these updates significantly enhance the technical precision and readability of our work.

**Reviewer #2, Concern #2:** Highlight Novelty in the Abstract: Introduce the unique aspects of your work at the beginning of the abstract to immediately convey its significance to the reader.

**Author response:**Thank you for your insightful suggestion regarding the clarity of the abstract. In response, we have revised the abstract to clearly introduce the novel aspects of our work at the beginning, specifically emphasizing the development of two novel deep learning models—SENARX and GA-LSTM—for concurrent electricity load and price forecasting. The revised abstract now highlights the unique integration of a sparse encoder and genetic algorithm-based optimization, as well as the models’ superior performance and robustness across multiple international electricity markets. This modification aims to immediately convey the significance and innovation of our contribution to the reader.

**Author action:** We have updated the manuscript in response to your valuable feedback. The changes are marked in red in the revised manuscript on page 1.

**Reviewer #2, Concern #3:** Hyphen Usage: Ensure consistent use of hyphens and dashes throughout the manuscript, adhering to standard formatting guidelines. Acronym Introduction: Define all acronyms upon their first occurrence to aid reader comprehension. Introduction Enhancements: Smooth Transitions: Refine the flow between paragraphs to create a cohesive narrative that guides the reader through the background and purpose of the study. Emphasize Relevance: Clearly articulate the importance of your research and its potential impact to engage the reader effectively. Abstract Content: Incorporate key results and findings into the abstract to provide a concise summary of your study's outcomes. Citation Accuracy: Replace any placeholder symbols (e.g., question marks) with appropriate citations to maintain academic integrity.

**Author response:** Thank you for your detailed and constructive feedback. We have carefully addressed each of your recommendations as follows:

- **Hyphen Usage:** We reviewed the manuscript and ensured consistent usage of hyphens and en dashes according to standard formatting and typographic guidelines.
- **Acronym Introduction:** All acronyms are now defined upon their first appearance to enhance clarity and aid reader comprehension (e.g., GA for Genetic Algorithm, LSTM for Long Short-Term Memory).
- **Introduction Enhancements:**
  - *Smooth Transitions:* We revised the structure of the introduction to improve the flow between paragraphs, ensuring a cohesive and logical narrative.
  - *Emphasize Relevance:* We now more clearly state the motivation, significance, and broader implications of our study in the introductory section to better engage the reader.
- **Abstract Content:** The abstract has been updated to include key results and performance metrics, providing a clear and concise overview of the study’s contributions and outcomes.
- **Citation Accuracy:** All placeholder symbols and missing references have been replaced with appropriate and accurate citations to ensure academic integrity.

**Author action:** Thank you for your valuable feedback. These revisions are reflected throughout the revised manuscript and are highlighted in red for clarity. We appreciate your constructive suggestions, which have helped improve the clarity and quality of the manuscript.

**Reviewer #2, Concern #4:** Avoid First Person: Utilize third-person perspective to maintain a formal academic tone. Eliminate Bullet Points: Present information in well-structured paragraphs instead of bullet points to align with scholarly writing conventions. Consistency in Text Formatting: Ensure uniform font size and style throughout the document to provide a professional appearance. Grammar and Syntax: Conduct a thorough review to correct grammatical errors and improve sentence structure for clarity and readability. Explanation of Key Concepts: Provide a brief overview of "big data" to ensure all readers have a foundational understanding of the term. Data Set Disclosure: Clearly describe the data set used in your study, including its source and any relevant characteristics.

**Author response**:The authors appreciate the reviewer’s insightful suggestions, which have been carefully implemented to enhance the manuscript's academic quality. To maintain a formal scholarly tone, the use of first-person pronouns has been removed throughout the document, with all text revised to reflect a third-person perspective. Bullet points have been eliminated and replaced with coherent, well-structured paragraphs to align with standard academic writing practices. A comprehensive review of the manuscript was conducted to ensure consistency in font size and style, resulting in a more professional and uniform presentation. Furthermore, grammatical and syntactical corrections have been made throughout the text to improve clarity and readability. To support readers who may be unfamiliar with the term “big data,” a brief explanatory statement has been added in the introduction, outlining its definition and relevance in the context of energy forecasting. Additionally, the manuscript now includes a clear and detailed description of the dataset used in the study, specifying its source, coverage period, and key characteristics to improve transparency and reproducibility.

**Author action:**Thank you for your valuable feedback. We have revised the manuscript accordingly. These revisions can be found on pages 2, 3, and 5 of the revised manuscript and are highlighted in red for easy identification. We appreciate your guidance and believe the changes enhance the clarity and presentation of the work.

**Reviewer#2, Concern#5:** Table and Figure Presentation: Introduce Tables: Offer context and explanation before presenting Table 1 to prepare the reader for the information it contains. Discuss Results: Include a narrative that interprets and discusses the results shown in tables and figures, enhancing their contribution to your study's findings. Figure Descriptions: Provide detailed explanations following each figure to elucidate their relevance and insights. Remove Grid Lines: Eliminate unnecessary grid lines from figures to improve visual clarity. Enhance Legibility: Ensure that all figure legends and labels are legible, particularly in Figures 4 and 6. Clarify Axis Labels: Review and standardize axis labels, such as "(hr-end)" in Figure 4, for consistency and clarity. Differentiate Data Series: Modify Figure 5 to clearly distinguish between actual and predicted data, possibly by using varied line styles or colors. Table Formatting: Enlarge Table 2 to enhance readability and ensure it is easily interpretable. Proximity of Discussions to Visuals: Position discussions of figures and tables adjacent to their respective visuals to facilitate immediate comprehension and reference.

**Author response:** The authors sincerely thank the reviewer for the constructive feedback on the presentation of tables and figures. In response, several improvements have been made to enhance the clarity and effectiveness of visual elements in the manuscript. Contextual introductions have been added prior to each table, particularly before Table 1, to prepare the reader for the data and its relevance. Each figure and table is now followed by an explanatory narrative that interprets the results and highlights their contribution to the study’s findings. To improve visual clarity, unnecessary grid lines have been removed from all figures. The legibility of figure legends, labels, and titles—especially in Figures 4 and 6—has been improved by increasing font size and adjusting layout. Axis labels such as “(hr-end)” in Figure 4 have been standardized and clarified to ensure consistency. Figure 5 has been modified to distinctly differentiate between actual and predicted data using contrasting line styles and color schemes. Additionally, Table 2 has been enlarged and reformatted for enhanced readability and ease of interpretation. To support immediate comprehension, the discussion of each visual element has been repositioned to appear adjacent to the corresponding figure or table in the manuscript. These revisions are clearly marked in red in the revised version, primarily on pages 6 through 10.

**Author action:**Thank you for your valuable feedback. These revisions are clearly marked in red in the revised version, primarily on pages 1 through 22. We appreciate your suggestion, which helped improve the clarity and readability of our work.

**Reviewer#2, Concern#6:**  Additional Comments on the Conclusion: The conclusion provides valuable insights into the performance of SENARX and GA-LSTM, but it could benefit from a few enhancements: Clarify Key Findings: Start by clearly summarizing the key results first—such as the superior performance of SENARX and GA-LSTM in terms of accuracy and convergence speed—before diving into specific metrics. Address Broader Implications: Consider briefly discussing the broader implications of your findings. For example, how might the superior performance of these models influence real-world smart grid applications? What challenges may arise in practical deployment? Comparison with Existing Literature: It would be beneficial to briefly mention how your results compare with previous work in the field. How do your models advance the state-of-the-art? Acknowledge Limitations: Briefly mentioning any limitations (e.g., dataset constraints or potential generalization issues) would provide a more balanced perspective. Future Work: Including a sentence or two on future directions, such as potential adaptations of your models for other forecasting problems, could add depth to the conclusion.

**Author response:** The authors appreciate the reviewer’s insightful suggestions to strengthen the conclusion section. In response, the conclusion has been revised to begin with a concise summary of the key findings, specifically highlighting the superior performance of the proposed SENARX and GA-LSTM models in terms of forecasting accuracy and convergence speed. To enhance the contextual impact, a discussion on the broader implications of the results has been included—emphasizing how improved accuracy and robustness can support more reliable and responsive smart grid operations in real-world scenarios. A brief comparative statement has also been added to position the proposed models relative to existing literature, illustrating how this work advances the state-of-the-art in short-term load and price forecasting. Moreover, the conclusion now acknowledges certain limitations of the study, such as the potential influence of dataset characteristics and challenges in generalizing the models across diverse energy markets. Finally, future directions have been incorporated, including potential model adaptations for forecasting under extreme market conditions or integration with renewable energy prediction frameworks.

**Author action:**Thank you for your valuable feedback. These revisions are clearly marked in red in the revised version, primarily on pages 1 through 22. We appreciate your suggestion, which helped improve the clarity and readability of our work.

**Reviewer#2, Concern#6:**  Clarify "Ideal Candidates": The phrase "making them ideal candidates for real-time smart grid applications" could be made more specific. For instance, describe how these models outperform others in real-time scenarios or in specific use cases where their benefits are most noticeable. Simplify Technical Jargon: Consider briefly explaining terms like "MAPE," "GA-LSTM," and "SENARX" for readers who may not be as familiar with these technicalities. Polish Flow: Consider refining the flow of the conclusion, such as moving the discussion of noise sensitivity and robustness before the convergence speed for better readability and logical progression. By addressing these points, the conclusion will provide a more well-rounded perspective, emphasizing the significance of your work and its potential impact on real-world applications. Implementing these recommendations will significantly improve the manuscript's quality and its engagement with the audience.

**Author response:** We thank the reviewer for their constructive suggestions to enhance the clarity and impact of the conclusion. In the revised manuscript (highlighted in red on page 13), we have addressed the following points:

- **Clarified “Ideal Candidates”** by specifying the advantages of SENARX and GA-LSTM in real-time smart grid environments, such as their lower computational cost, faster convergence, and superior accuracy under volatile conditions.
- **Simplified Technical Jargon** by briefly defining terms such as Mean Absolute Percentage Error (MAPE), GA-LSTM (Genetic Algorithm optimized Long Short-Term Memory), and SENARX (Sparse Encoder Nonlinear Autoregressive model with Exogenous inputs).
- **Refined the Logical Flow** by discussing the models' robustness to noise before addressing their convergence speed, resulting in a smoother narrative progression.

These changes collectively improve the readability and accessibility of the conclusion while emphasizing the practical significance and potential deployment of our models in real-world smart grid forecasting tasks.

**Author action:**Thank you for your valuable feedback. These revisions are clearly marked in red in the revised version, primarily on pages 1 through 22. We appreciate your suggestion, which helped improve the clarity and readability of our work.
